# Bile Acids Activated Receptors in Inflammatory Bowel Disease

**DOI:** 10.3390/cells10061281

**Published:** 2021-05-21

**Authors:** Michele Biagioli, Silvia Marchianò, Adriana Carino, Cristina Di Giorgio, Luca Santucci, Eleonora Distrutti, Stefano Fiorucci

**Affiliations:** 1 Department of Medicine and Surgery, University of Perugia, 06132 Perugia, Italy; silvia4as@hotmail.it (S.M.); adriana.carino@hotmail.it (A.C.); cristi.digiorgio@gmail.com (C.D.G.); stefano.fiorucci@unipg.it (S.F.); 2 Azienda Ospedaliera di Perugia, 06132 Perugia, Italy; lsant6461@libero.it (L.S.); eleonoradistrutti@katamail.com (E.D.)

**Keywords:** bile acids, intestinal microiota, FXR, GPBAR1, RORγt, intestinal immunity

## Abstract

Once known exclusively for their role in nutrients absorption, bile acids have emerged as signaling molecules, generated from cholesterol breakdown, acting on several immune cells by activating a variety of receptors including the G protein-coupled bile acid receptor 1 (GPABR1 or TGR5), the Farnesoid-X-receptor (FXR) and, as recently discovered, the retinoid-related orphan receptors (ROR)γt. GPBAR1, FXR, and RORγt are highly expressed in cells of the innate and adaptive immune system (i.e., dendritic cells (DCs), macrophages, innate lymphoid 3 cells (ILC3s), and T helper 17 (Th17) lymphocytes) and plays an important role in regulating intestinal and liver immunity, highlighting a role for various bile acid species in regulating immune responses to intestinal microbial antigens. While primary bile acids are generated from the cholesterol breakdown secondary bile acids, the GPBAR1 ligands, and oxo-bile acids derivatives, the RORγt ligands, are generated by the intestinal microbiota, highlighting the potential of these bile acids in mediating the chemical communication between the intestinal microbiota and the host. Changes in intestinal microbiota, dysbiosis, alter the composition of the bile acid pool, promoting the activation of the immune system and development of chronic inflammation. In this review, we focus on the molecular mechanisms by which an altered bile acid signaling promotes intestinal inflammation.

## 1. Introduction

Bile acids are amphipathic molecules essential for the absorption of lipids in the intestine. However, bile acids are also signaling molecules regulating a number of physiologically relevant processes by activating a group of G-protein-coupled and nuclear receptors collectively known as “Bile acid-activated receptor “(BARs) [1,2]. BARs are widely expressed by cells of innate immunity and their activity is mostly inhibitory in nature, suggesting that these receptors might be an essential component of a counter-regulatory system that promotes the development of a *tolerogenic* state of the intestinal immune system in face of the antigenic load generated by intestinal microbiota. For this reason, bile acids and their receptors represent an interesting therapeutic target for the development of new therapies in inflammatory bowel diseases (IBD) [2,3,4,5]. Here, we will review the role of bile acids as essential modulators of the intestinal immune system, and how dysregulation of bile acid signaling might have mechanistic relevance in the development of IBD. Finally, we will provide some insights on how BARs may represent an interesting therapeutic target in the development of novel therapies for IBD [6,7].

## 2. Bile Acids Metabolism

Bile acids are a large family of atypical steroids generated in the mammalian body by the interaction of multiple enzymes provided by the liver and intestinal microbiota. While similar to other steroid hormones, bile acids are primarily derived from cholesterol, they have a peculiar chemical structure. Indeed, in contrast to cholesterol, bile acids are amphipathic molecules with a hydrophobic side (β face) and a hydrophilic side (α face). This amphipathic structure gives them detergent properties that are essential for solubilizing lipids in the micelles, facilitating emulsification and absorption of dietary lipids and fat-soluble vitamins [8]. Primary bile acids, cholic acid (CA) and chenodeoxycholic acid (CDCA), are generated in the human liver directly from cholesterol breakdown [9] and after conjugation with glycine or taurine, are transported through the bile duct into the intestine where they undergo a series of modifications operated by the intestinal microbiota to generate secondary (or degenerated) bile acids: dexoycholic acid (DCA) and litocholic acid (LCA) [2,9,10,11,12]. The synthesis of primary bile acids is carried out by hepatocytes through two pathways known as the neutral (or classical) and the acidic (or alternative) pathway. The classical pathway is responsible for the synthesis of approximately 90% of primary bile acids and produces the same amount of CA and CDCA [12]. In the classic pathway, the first and rate-limiting enzyme is the cholesterol-7α-hydroxylase (CYP7A1) which irreversibly converts cholesterol into 7α-hydroxy-cholesterol. This intermediate is then converted to 7α-hydroxy-4-cholesten-3-one by 3β-hydroxy-Δ5-C27-steroid oxidoreductase (HSD3B7), an intermediate metabolite that could be used for generating both CA and CDCA in the classic pathway. The ratio between the two primary bile acids is determined by the sterol 12a-hydroxylase (CYP8B1), which is required for CA synthesis. Conversely, the alternative (or acidic) pathway starts with transformation of cholesterol to 27-hydroxy-cholesterol by sterol 27-hydroxylase (CYP27A1), followed by hydroxylation on ring B, metabolized by oxysterol 7α-hydroxylase (CYP7B1), and side-chain modification which produces CDCA. The alternative pathway generates only CDCA and contributes 10% of total bile acid pool generated in the liver [13,14]. Before secretion into bile ducts, the primary bile acids are conjugated with glycine or taurine in position C-24 by the bile acyl CoA synthetase and bile acid-CoA amino acid N-acyltransferase (BAAT), resulting respectively in the tauro-CA (TCA) and tauro-CDCA (TCDCA), and glyco-CA (GCA) and glycol-CDCA (GCDCA). In the human liver, CA and CDCA are amidated with glycine and taurine at a ration about 3:1. In contrast to human, in mice, approximately 95% of primary bile acids are tauro-conjugated. The amidated derivatives of primary bile acids are indicated as bile salts and are secreted into the bile ducts and transported to the intestine where they undergo additional biotransformation by the intestinal microbiota.

## 3. BAs and Intestinal Microbiota

In the distal ileum, conjugated bile acids are reabsorbed through Apical Sodium Dependent Bile Acid Transporter (ASBT) expressed on the apical membrane of enterocytes and transported back to the liver through the entero-hepatic circulation. In contrast, unconjugated bile acids escape uptake through ASBT and enter the colon where they undergo further metabolism by intestinal microbiota to generate secondary bile acids. Microbial deconjugation (i.e., removal of the glycine or taurine conjugate) prevents active reuptake from the small intestine and is carried out by bacteria with bile salt hydrolase (BSH) activity. Metagenomic analyses demonstrated that functional BSH is present in all major anaerobic bacterial divisions and archaeal species in the human gut including members of *Bacteroides*, *Clostridium*, *Lactobacillus*, and *Bifidobacteria*. In fact, BSH is enriched in the gut microbiota compared with other microbial ecosystems and is associated with increased resistance to bile toxicity. Subsequently, the enzyme 7α-dehydroxylase expressed by *Clostridium* and *Eubacterium* performs the 7α-dehydroxylation on ring B that converts CA in deoxycholic acid (DCA) and CDCA in lithocholic acid (LCA) called secondary (or degenerated) bile acids. In humans, the bile acid pool consists of CA (≈40%), CDCA (≈40%), DCA (≈20%), with a glycine over taurine conjugation ratio of 3–1 [2,15,16].

Other bacteria contribute to different metabolic biotransformations: the C7 β-epimerization of CDCA operated by *Escherichia*, *Clostridium*, *Bacteroides*, and *Eubacerium* originates the 7β-epimer of CDCA known as ursodeoxycholic acid (UDCA).

One important species-specificity of bile acid metabolism takes place in mice. Indeed, in contrast to humans, mice only generate CA from cholesterol, since CDCA in the rodent liver is transformed into α- and β-muricholic acid (MCA) by the enzyme CYP2C70 present only in mice and not in humans [9,17]. For this reason, α- and β-MCA are primary bile acids in mice. In the intestine, α- and β-MCA are metabolized into murideoxycholic acid (MDCA). Omega-MCA (ωMCA) is a major metabolite of βMCA and is formed by 6β-epimerization. Other metabolites of βMCA are hyodeoxycholic acid (HDCA), formed by βb-epimerization and additional βb-dehydroxylation, and hyocholic acid (HCA), formed by 6β-epimerization and further 7β-epimerization [2,9,10,11,12].

In addition, the intestinal microbiota generates other bile acid derivates: 3-, 7-, and 12-oxo-bile acid which represent about 20–30% of bile acid metabolites produced by gut microbiota in the colon. The generation of oxo bile acids is catalyzed by the 3α, 7α, and 12α-hydroxysteroid dehydrogenases (HSDHs), which are mainly found in *Clostridium clusters XIVa* (i.e., C. scindens, C. hiranonis, and C. hylemonae), *IV*, and *XI* [18].

Recently, new microbiome-conjugated bile acids with tyrosine, phenylalanine, and leucine have also been identified, which give rise respectively to tyrosocholic acid, phenylalanocholic acid, and leucocholic acid [19]. These new bile-acid conjugates were found in humans and were enriched in patients with inflammatory bowel disease or cystic fibrosis and early data indicate that they are FXR receptor antagonists [19].

The large majority of these deconjugated primary bile acids and secondary bile acids that reach the terminal ileum are reabsorbed by the intestinal epithelial cells (IEC) and transported back to the liver through the portal vein, completing a cycle in the “entero-hepatic circulation” [11,20,21].

## 4. Bile Acids Activated Receptors (BARs) and Intestinal Immunity

Bile acids are the largest family of steroidal mediators found in mammalians, and as mentioned above, have been identified as the physiological ligands GPCR and nuclear receptors, mainly expressed in the entero-hepatic system and in immune cells [16,22]. The two best characterized receptors belonging to the BAR family are the Farnesoid-X-receptors (FXR), and the G protein bile acid activated receptor (GPBAR)-1, also known as Takeda G-protein-coupled receptor 5 (TGR5) [23,24,25,26,27]. FXR, originally described in 1995 and de-orphaned in 1999, is a nuclear transcription factor activated by primary bile acids. Moreover, 6α/βMCA bile acid, the two main bile acids found in mice in addition to TCA, acts as an FXR antagonist [28,29]. GPBAR1 is a seven-transmembrane G-protein coupled receptor, discovered in 2002, mainly activated by secondary bile acids. These two receptors are activated by bile acids at relatively low concentrations, but bile acids at high concentrations also activate other both membrane and nuclear receptors. Among the membrane receptors activated by bile acids, we find the sphingosine 1-phosphate receptor (S1PR2) [30], which binds LCA, the muscarinic receptor M2 and M3, activated by DCA and LCA, the formyl peptide receptors (FPR), of which CDCA is an antagonist [31], and vascular endothelial growth factor receptor (VEGF-R), activated by CDCA. As for nuclear receptors, bile acids also activate the constitutive androstane receptor (CAR, also known as NR1H3) [32], the pregnane-x-receptor (PXR, also known as NR1H2) [33], activated by CDCA, LCA, and DCA, and the vitamin D receptor (VDR, also known as NR1H1), activated by LCA and DCA [34]. Moreover, hyodeoxycholic acid (HDCA or Hyo-DCA), present in high concentrations in patients with cholestasis, activates the liver-X-receptor α and β (LXRα/β, NR1H3) [35]. The oxo-bile acids are gaining growing attention in the last years for the ability of some 3-oxo-bile acids to bind to the retinoid-related orphan receptor (ROR)ɣt on which act as antagonists [36].

**Figure 1:**
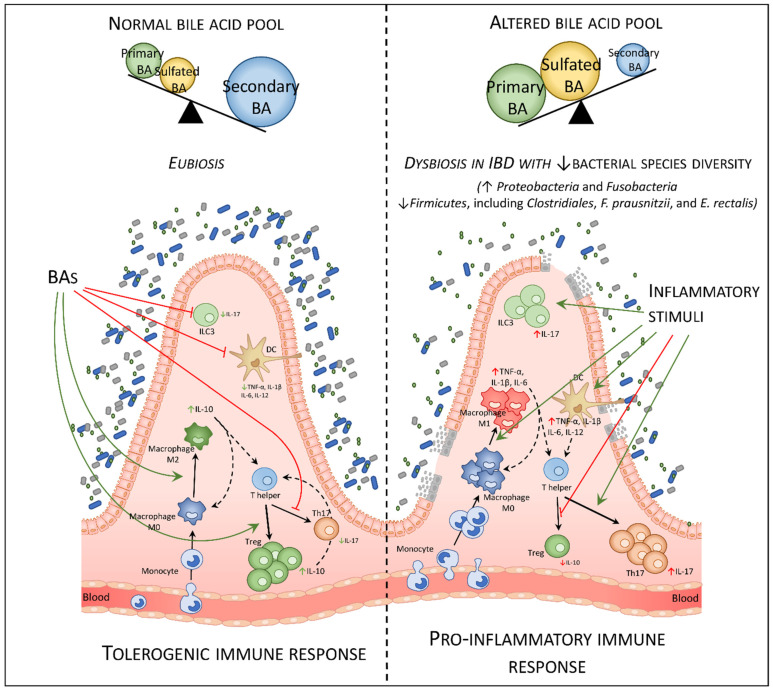
Microbiota, bile acid pool, and regulation of intestinal immunity. In a healthy condition, the majority of bile acids are actively reabsorbed by the enterocytes by apical transporter ASBT, and are transported back to the liver through the portal blood, thus limiting BA loss through feces to 3%–5% of daily secreted BAs. BAs reaching the colon are metabolized by the intestinal microbiota which transforms primary bile acids into secondary bile acids and other derivatives which therefore make up the majority of the bile acid pool. BAs through agonism on FXR and GPBAR1 and antagonism on RORγt (inverse agonism) regulate the immune system by inducing a tolerogenic response. The action on these receptors induces the polarization of macrophages and helper T cells towards an anti-inflammatory phenotype, respectively macrophages M2 and Treg, with the up-regulation of IL-10 production, and inhibits the activation of DCs, ILC3, and Th17 by reducing the production of pro-inflammatory cytokines (i.e., IL-6, IL-1β, TNF-α, and IL-17). In patients with IBDs, the alterations of the intestinal epithelium reduce the reabsorption of bile acids and therefore increase the quantity of bile acids that are eliminated with the feces. Furthermore, patients with IBDs have a dysbiosis of the intestinal bacterial flora with a decrease in bacterial species diversity, which strongly decreases the enzymatic capacity of the microbiota, resulting in a lower ability to metabolize primary bile acids into secondary bile acids and other derivatives. After breach of the epithelial barrier, or pathogenic invasion, molecules like LPS activate macrophages, DCs, and ILC3, inducing the production of pro-inflammatory cytokines, IFN-γ, TNF-α, IL-6, IL-1β, and IL-17, with an increase in the polarization of M0 macrophages toward a pro-inflammatory M1 phenotype. M1 macrophages and activated DCs therefore induce production of effector T cells (i.e., Th17) and up-regulation of the expression of chemokine CCL2 in the colon, which recalls more monocytes from the blood to the lamina propria of the colon.

BARs widely expressed receptors and are found in different cells of the gastro-intestinal tract: intestinal epithelial cells (FXR and GPBAR1), intestinal muscle and neurons (GPBAR1), biliary cells (FXR and GPBAR1), hepatocytes (FXR), liver sinusoidal cells (FXR and GPBAR1), and liver and intestinal endothelial cells (FXR and GPBAR1) [11,37,38]. Moreover, both receptors are highly expressed in cells of the innate immune system such as monocytes/macrophages cells, dendritic cells (DCs), natural killer (NK) and NKT cells [26,39,40,41,42,43,44,45]. In contrast, the cells of the adaptive immune system express low levels of both FXR and GPBAR1 [43]. Activation of BARs in macrophages, DCs, and NKT cells results in many regulatory functions which collectively induce a tolerogenic immune response in the intestine and liver essential for the maintenance of immunological tolerance towards the continuous flow of dietary xenobiotics and antigens generated by the intestinal microbiota (Figure 1).

A separate mention should be made of the RORγt receptor which, as already mentioned, binds oxo-bile acids as antagonists, and which is highly expressed in a subgroup of the innate lymphoid cells (ILC), the ILC3 cells, and in the T helper lymphocytes Th17 [36].

### 4.1. FXR

**Figure 2:**
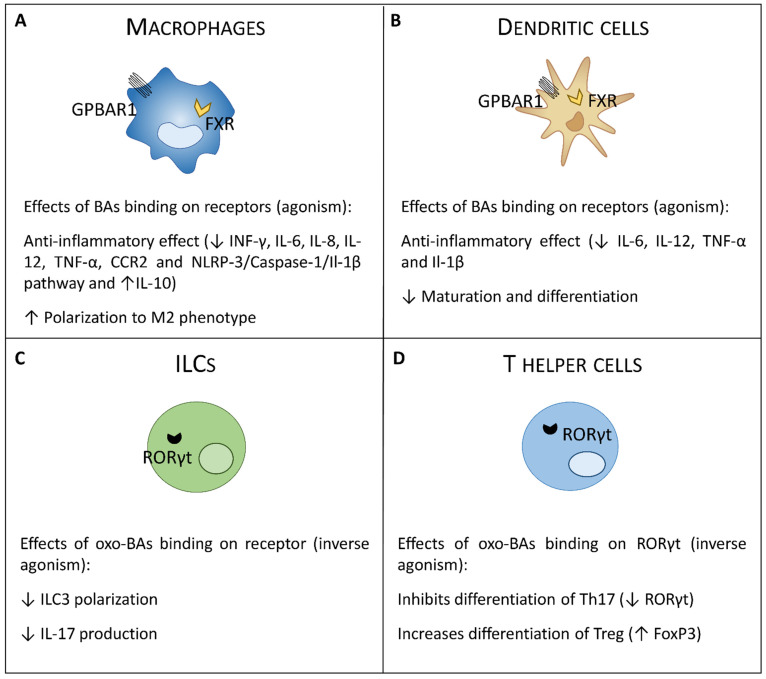
Expression and functional role of G-protein bile acid receptor 1 (GPBAR1), Farnesoid-X-receptor (FXR) and RORγt in cells of immunity. Macrophages and dendritic cells (DCs) express GPBAR1 and FXR. T helper cells and ILCs express RORγt. (**A**) In macrophages, activation of these receptors by bile acids induces a polarization toward the anti-inflammatory M2 phenotype with an upregulation of IL-10 and a downregulation of the pro-inflammatory cytokines. (**B**) Bile acids, on the other hand, act on the DCs, down-regulating the production of TNF-α and IL-12 and their maturation and differentiation. (**C**,**D**) Recently, it was shown that oxo-bile acid derivatives, specifically the 3-oxo-LCA, can bind RORγt by acting as an inverse agonist, decreasing the production of IL-17 in T helper cells and ILCs by reducing the polarization towards the Th17 and ILC3 subtypes.

The immunomodulatory action exerted by FXR on monocytes and macrophages was originally demonstrated in 2009 [39]. In these cells, FXR modulates the expression of multiple genes involved in inflammation through both SHP-dependent and independent mechanisms [42,46]. The expression of atypical nuclear receptor small heterodimer partner (SHP) is generally used to confirm an FXR activation because FXR directly regulates SHP expression [47]. SHP does not have the DNA binding domain and therefore exerts its activity through protein–protein interactions by acting as a co-repressor, facilitating the recruitment of other co-repressors on the promoter of FXR target genes [48]. Yang et al. [49] showed that SHP stabilizes the binding of the inhibitory complex on the promoter of the chemokine (C-C motif) ligand (CCl) 2 by inhibiting the recruitment of the NF-kB p65 subunit by decreasing the expression of this gene. Another important mechanism that mediates the immune-regulatory activity of FXR in macrophages is SHP-independent. FXR, after activation by specific ligands like primary bile acids, is recruited directly on the promoter of several pro-inflammatory genes such as iNOS, TNF-α, and IL-1β on which stabilizes the nuclear receptor corepressor 1 (NCor1) complex. At the basal state, the NCor1 complex is bound on the promoter of these genes, preventing the binding of NF-kB and keeping them in a state of transcriptional inactivity [41]. The activation of toll like receptor-4 (TLR-4) causes the release of NCor1 from the promoters, allowing the transcriptional activation of these genes [39]. On the contrary, in the presence of an agonist, such as obeticholic acid (OCA, also known as INT-747), FXR is recruited to the iNOS and IL-1β promoters and stabilizes the NCoR1 complexes on the promoters of these two genes, causing a trans-repression [42] (Figure 2A).

Another anti-inflammatory mechanism promoted by FXR involves the regulation of inflammasomes, including NLRP1, NLRP3, NLRC4, AIM2 family members, a class of cytoplasmic multi-protein complexes that sense endogenous and exogenous pathogen-associated or danger-associated molecular patterns (PAMPs and DAMPs) [50]. The canonical inflammasomes are made up by a nucleotide-binding domain and leucine-rich repeat-containing proteins (NLRs) or AIM2, adaptor protein ASC, and caspase-1, a protease that mediates the cleavage of precursors of cytokines of the IL-1 family, i.e., IL-1β and IL-18. NLRP3 is one of the most comprehensively characterized inflammasomes and its excessive activation has been detected in different inflammatory disorders.

FXR function as a negative modulator of NLRP3 assembly through a physical interaction with with NLRP3 and caspase 1. In addition to these mechanisms, SHP has been shown to prevent NLRP3 formation [51,52] (Figure 2A). However, it should be remembered that high concentrations of bile acids, that are usually observed only in patients/models of obstructive cholestasis induce activation of the inflammasome [53].

FXR is expressed also by DCs and NKT cells. Two different studies have shown that FXR activation reduces the differentiation and activation of intestinal DCs by down-regulating TNF-α expression and alleviating the severity of colitis in mouse models. Furthermore, activation of FXR inhibits the differentiation of CD14+ monocytes into mature DCs [54,55] (Figure 2B). The effect of FXR activation in NKT cells has been demonstrated only in the liver. In this study, we have shown that obeticholic acid (INT-747) inhibited the influx of NKT cells and the ability of these cells to produce osteopontin in rodent models of acute hepatitis [42,56,57].

In summary, FXR exerts counter-regulatory activity on monocytes/macrophages, DCs, and NKT cells through SHP-dependent and independent mechanisms that often involve negative regulation of the NF-kB pathway.

### 4.2. GPBAR1

There is growing interest in GPBAR1 pharmacology and on its immune-regulatory effects [10,14]. The regulation of monocytes and macrophages effector functions by GPBAR1 has been demonstrated originally by Kawamata et al. [27]. Moreover, other innate immune cells such as DCs and NKT cells express the receptor [40,58,59]. Activation of GPBAR1 in macrophages modulates multiple pathways. The binding of GPBAR1 with specific natural or synthetic agonists activates the protein kinase A (PKA) which induces the phosphorylation of CREB, favoring its binding on the promoter of specific target gens. pCREB acts both as an inducer of the transcription of some anti-inflammatory genes such as IL-10 [43], and as an inhibitor of the transcription of pro-inflammatory genes such as IL-1β or TNF-α by reducing the activity of NF-kB on the promoter of these genes [60]. Thanks to this dual activity, the activation of GPBAR1 shift colonic macrophages from M1 pro-inflammatory phenotype to a M2 anti-inflammatory phenotype, relieving colitis in mouse models of the disease [2,43]. Furthermore, the agonism of GPBAR1 reduces the influx of macrophages into the lamina propria of the colon after an inflammatory stimulus [43], suggesting that the differentiation of monocytes toward a pro- or anti-inflammatory phenotype in the colon is regulated by intestinal GPBAR1, in addition to FXR [1]. These data were also confirmed in vitro in human macrophage studies in which the activation of GPBAR1 down-regulated the expression of IFN-γ, IL-1β, IL-6, and TNF-α while inducing the expression of IL-10 [59] (Figure 1 and Figure 2A).

Intestinal DCs also express GPBAR1. These cells sense pathogens and direct the appropriate immune response, ensuring the maintenance of tissue homeostasis. Activation of GPBAR1 in DCs attenuates the bias towards the pro-inflammatory phenotype that produces IL-12 and TNF-α by promoting the polarization towards a tolerogenic phenotype that produces low levels of IL-12 [59,61] (Figure 2). NKT cells express both FXR and GPBAR1 but the role of activating also GPBAR1 in these cells has been investigated only in the liver. We demonstrated in two mouse models of acute hepatitis induced by concanavalin A (Con A) or α-galactosyl-ceramide (α-GalCer), that the activation of GPBAR1 with a synthetic selective ligand alleviates the disease by counteracting the polarization of NKT towards the pro-inflammatory subgroup NKT1 while inducing bias toward a NKT10, a regulatory, IL-10 secreting, subset of NKT cells [44]. These data suggest that a similar effect may also be exerted by GPBAR1 on intestinal NKT cells but further investigations are needed.

Like FXR, GPBAR1 also exerts a counter-regulatory effects on the activation of the NLPR3 inflammasome [50,53]. Activation of GPBAR1 by secondary bile acid DCA and LCA cause a GPBAR1-cAMP-PKA-dependent ubiquitination of NLRP3, thus inhibiting its activation [50,53,62].

## 5. RORγt

The retinoid-related orphan receptors (RORs) are a family of three nuclear receptors: RORα, β, and γ. The RORα, β, and γ genes have been mapped to the human chromosome 15q22.2, 9q21.13, and 1q21.3, respectively. RORγ generates two different isoforms: RORγ1 and RORγt (or γ2) encoded by the gene RORC. However, while RORγ1 co-regulates (often in co-operation with RORα) the transcription of several circadian and metabolic genes in adipose tissues and liver, expression of RORγt is restricted to specific subsets of immune cells of lymphoid lineage, i.e., T helper 17 (Th17) cells, innate lymphoid 3 cells (ILC3s), and γδ T cells [63,64,65,66,67,68] (Figure 1 and Figure 2C, D). Recently, in regards to the BARs family, growing interest is turning to RORγt, because in recent years, it has been shown that RORγt can bond some oxo-derivatives of bile acids, in particular, the 3-oxo-LCA as an inverse agonist. The action of oxo bile acid derivatives on the RORγt receptor is very interesting because this receptor is expressed by other cells of the immune system than those expressing BARs, detected in myeloid cells, thus expanding the action of bile acids also on adaptive immune system.

In CD4+ T cells, RORγt is required for Th17 cells differentiation and for IL-17 production by these T cells and by type 3 lymphoid cells (ILC3) [69]. In the intestine, RORγt appears essential to maintain homeostasis with the symbiotic microbiota. Although RORs are considered orphan nuclear receptors, various oxysterols, and in particular, the cholesterol precursor, the 25-hydroxycholesterol, activate RORγt [70]. In 2019 [71] and then in 2020 [36], two independent research groups demonstrated that the BAs derivates can bind RORγt by acting as an inverse agonist. 

Hang S. et al. demonstrated that 3-oxo-LCA inhibited the differentiation of Th17 cells by directly binding to the RORγt and that the administration of 3-oxoLCA to mice reduced Th17 cell differentiation and increased Treg cell differentiation, in the intestinal lamina propria, relieving colitis in the mouse CD4^+^ T cell transference model.

Both groups demonstrated that binding of RORγt to 3-oxo-LCA decreased IL-17 production and Th17 cell numbers by attenuating intestinal inflammation in a mouse model of colitis [72,73]. Together, these findings highlight a potential role for RORγt inverse agonists or antagonists in regulating inflammation at the interface of intestinal microbiota and host immune system.

## 6. Bile Acids Signaling in IBD

IBDs are chronic diseases caused by a dysregulation of the immune response to luminal antigens in genetically predisposed individuals. The two main clinical manifestations are Crohn’s disease (CD) and ulcerative colitis (UC). The prevalence of these conditions is growing worldwide and becoming an emerging health problem everywhere. Changes in the composition of the gut microbiota are considered one of the main triggers of IBD but the molecular mechanisms and mediators involved are not yet fully understood. In this context, bile acids generated at the interface between the host and the intestinal microbiota are attracting increasing interest. Several studies over the years have investigated the composition of the bile acid pool in patients with IBD. These studies have shown that a reduction in the bile acid pool is present in IBD patients only when the disease involves both the ileum and the colon. Vantrappen et al. provided evidence as early as 30 years ago that CD patients, but not UC patients, show a reduction of the bile acid pool size. Moreover, in that study, they demonstrated an inverse correlation between the size of the bile acid pool and the Colitis Disease Activity Index (CDAI) [74]. Similar results were reported in 1982 using a cohort of CD patients [75]. In the later study, patients with ileal dysfunction were characterized by an increased turnover of bile acids and a severe loss of CA that correlates to the extent of ileal disease. These changes occurred only in CD with ileocolic involvement [75]. A decreased excretion of secondary bile acids has been detected also in UC and attributed to a reduced transit time (diarrhea), reduced fecal pH, and impaired 7-alpha-dehydroxylase activity [76,77,78,79]. Over the years, several other studies have confirmed that a bile acid malabsorption occurs in IBD patients with ileocolic disease. Of relevance, not only a bile acid depletion occurs in CD patients with ileocolic disease, but also the composition of bile acid pool changes in patients with UC during the diseases’ flare, with an increased excretion of conjugated bile acids and a decreased excretion of secondary bile acids. In addition, an increase in 3-OH-sulfate bile acid was observed in patients with active IBD [80]. In a recent study, Franzosa et al. [81] demonstrated that patients with active IBD have a reduction in the fecal content of DCA and LCA (secondary bile acids) associated with a sharp increase in the content of primary bile acids. Taken together, these data support the notion that in patients with UC and ileocolic CD, an acute flare associates with bile acids malabsorption and increased excretion of primary bile acids. The reduction of secondary bile acids in the colon might be of pathogenic relevance, since secondary bile acids are the main ligands of GPBAR1 in the colon, and the GPBAR1, has discussed previously represses innate immunity activation. Additionally, these studies highlight the role of the intestinal microbiota as the possible cause of bile acid dis-metabolism. Data obtained in germ-free mice also support this concept, since mice raised in a germ-free condition show a robust decrease in the content of secondary bile acids, along with a significant increase in the content of conjugated bile acids and of 3-OH-sulfate bile acids, highlighting the essential role of the gut microbiota in deconjugation, dehydroxylation, and desulfation of bile acids [81] (Figure 1).

The composition of the intestinal microbiota is altered in a substantial proportion of IBD patients [82,83,84,85,86,87]. Dysbiosis, a condition characterized by a reduction in bacterial species diversity, accompanied by an increase in fungi and bacteriophages, has been documented in both UC and CD individuals. In individuals with IBD, there is an expansion of *Proteobacteria* and *Fusobacteria* with a reduction of *Firmicutes*, including *Clostridiales*, *F. prausnitzii*, and *E. rectalis* (Figure 1). The cause–effect relationship between dysbiosis and IBD is also supported by the positive results obtained in recent trials with probiotics in UC and fecal microbiota transplant (FMT), a procedure approved for the treatment of Clostridium difficile infections but not for IBD [88,89,90,91,92,93].

It is now clear that dysbiosis impacts the ability of the intestinal microbiota to regulate innate immunity in the intestine. Part of these dysfunctional communications between the altered microbiota and intestinal immune system are mediated by reduced generation of beneficial metabolites including short-chain fatty acids (SCFAs), tryptophan metabolites, and secondary bile acids and other bile acids derivatives such as the 3 and 7 oxo- bile acids [2]. Because secondary bile acids are preferential ligands for GPBAR1, and this receptor is highly expressed in the colon, one might speculate that these changes could further aggravate the immune dysfunction seen in IBD patients. This idea is also supported by the data obtained in Gpbar1^-/-^ mice. In fact, GPBAR1 knock-out mice develop spontaneous inflammation in the colon with advancing age and furthermore, when stimulated with inflammatory agents, they develop colitis much more severe than wild-type mice. On the other hand, the administration of GPBAR1 agonist reverts intestinal inflammation in mouse models of colitis with a strong increase of IL-10 production [43]. Further on, FXR knockout mice develop a subclinical inflammation with age and are more prone than their congenic counterparts to develop inflammation [1,16,94,95,96,97,98]. The mechanisms that support these immune-modulatory activities of FXR in cells of innate immunity involve both NF-KB-dependent and -independent pathways as discussed in previous paragraphs [39,55,99,100].

### 6.1. BAR501: Profiling of a Selective GPBAR1 Agonist in Preclinical Models of Colitis

In vivo studies have shown that ablation of the Gpbar1 gene in mice results in a phenotype characterized by molecular alterations of the structure of tight junctions between intestinal epithelial cells with an increase in expression and an abnormal subcellular distribution of zonulin 1, leading to an increased intestinal permeability [40]. Furthermore, when compared to their wildtype congenic counterparts, Gpbar1 knock-out mice show a higher basal level of inflammatory cytokines including *Il-1β* and *Tnf-α* [43]. The possible regulatory role of GPBAR1 in intestinal immunity was also confirmed by the analysis of surgical samples of the colon from CD patients. By immunohistochemistry analysis, we have shown that the expression of GPBAR1 increases in the colon of CD patients and this is due to the recruitment of CD14^+^ cells into the mucosa of these patients, especially in the granulomatous areas. These data suggest a role for GPBAR1 in the regulation of monocyte/macrophage trafficking to the intestine [40].

**Figure 3:**
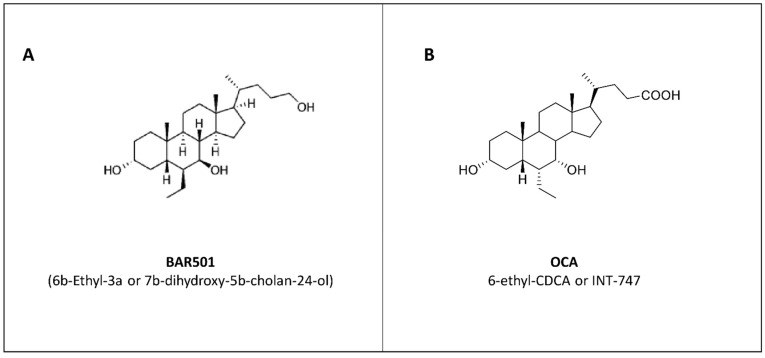
Chemical structures. Chemical structures of synthetic derivatives of BAs: (**A**) BAR501, selective agonist of GPBAR1, and (**B**) OCA, selective agonist of FXR.

Based on these preliminary data, we have focused our attention on the development of a selective agonist of GPBAR1. In 2014, by modifications on the cholane scaffold, we have obtained a compound christened BAR501 (6b-Ethyl-3a, 7b-dihydroxy-5b-cholan-24-ol), which is a selective agonist of GPBAR1, that activates the receptor with an EC_50_ = 1.03 μM (Figure 3A) [101].

The activity of BAR501 was extensively investigated in mouse models of colitis in the following years [43], in two mouse models of colitis induced by administration of oxazolone, which induces a UC-like Th2-mediated colitis, or by TNBS, which induces a CD-like Th1-mediated colitis. In these two models, exposure to BAR501 resulted in robust attenuation of signs and symptoms of colitis with beneficial effects on body weight loss, the Colitis Disease Activity Index (CDAI) and macroscopic and histologic features of colitis. These beneficial effects compared well with that exerted by glucocorticoid (dexamethasone) therapy. The attenuation of inflammation and immune dysfunction caused by BAR501 was supported by a shift in the polarization of colonic macrophages from a pro-inflammatory M1 phenotype (CD11b^+^Ly6C^−^CCR7^+^CD38^+^IL-6^+^), towards an anti-inflammatory M2 phenotype (CD11b^+^Ly6C^−^CCR7^−^Egr2^+^IL-10^+^). The shift was confirmed by the increased expression of specific markers for M2 phenotype such as *Egr2* and *C-myc*, and downregulation of *Cd38*, *Gpr18*, and *Fpr2*, which are signature genes for the M1 phenotype [43]. Importantly, although BAR501 effectively reduced the number of circulating monocytes, it failed to alter the ratio of Ly6C+/Ly6C2 cells, confirming that Ly6C expression per se does not affect the differentiation of monocytes toward a pro- or anti-inflammatory phenotype and that the differentiation of Ly6C+ monocytes, after they enter the tissues, depends on the organ microenvironment [102]. These data suggest that BAR501 might act at the level of the colon without dampening the systemic immune system unlike, for example, glucocorticoid therapy. The beneficial effects exerted by GPBAR1 agonism in these models were strongly associated with increased expression of IL-10 gene transcription in the intestine and enhanced secretion of IL-10 by lamina propria-derived macrophages. Treatment with BAR501 was also able to act indirectly on CD4^+^ T lymphocytes, despite the fact that these cells do not express GPBAR1. The modulation of the inflammatory response of CD4^+^ cells by the GPBAR1 agonist is mediated by the production of IL-10 by macrophage. In fact, IL-10 acts both on the macrophages themselves, inducing their polarization towards the M2 phenotype, and on CD4+ T cells by increasing the percentage of CD4^+^FoxP3^+^ Treg cells in the lamina propria of the colon. Together, these studies highlight a robust immune-modulatory activity of BAR501 and pave the way for further development in IBD.

### 6.2. Obeticholic Acid: Profiling of a Selective FXR Agonist in Colitis Models

*Fxr*_-/-_ mice spontaneously developed an altered expression of inflammatory mediators and increased intestinal permeability with age and developed a severe disease when challenged with DSS or TNBS [39,54,99]. We, and other research groups, have tested the use of a selective FXR agonist in mouse models of colitis. The first semi-synthetic agonist of FXR was obeticholic acid (OCA) (also known as 6-ethyl-CDCA or INT-747), which activates FXR with an EC_50_ of ≈100 nM (Figure 3B) [103,104,105,106]. In the TNBS-induced colitis mouse model, OCA was able to relieve colitis by reducing body weight loss, histological score, and expression of various inflammatory mediators (i.e., *i-Nos*, *Ifn-γ*, *Il- 1β*, *Il-6*, and *Tnf-α*) in a dose-dependent manner. OCA has been also tested in a chronic model of colitis (8 weeks), confirming its anti-inflammatory effects along with a robust anti-fibrotic activity, as illustrated by a reduction in the expression of *αSma*, *Fibronectin*, *a1-Col*, and *Tgf-β*. From a mechanistic standpoint, we have shown that in macrophages, the repression of *i-Nos* and *Il- 1β* exerted by OCA was due to the binding of FXR to the promoter of the two genes where it stabilizes the NCoR complex on the promoter of these genes [39]. The beneficial effects of OCA in models of colitis have been confirmed by others [54,99]. Gadaleta et al. showed that OCA significantly decreased the severity of DSS- and TNBS [54,99] e. The beneficial effects were completely abrogated in *Fxr^−/−^* mice, demonstrating that OCA improvement of colitis requires FXR. Furthermore, OCA has been shown to reduce intestinal permeability induced by DSS and TNBS, highlighting another potential benefit of FXR in regulating the integrity of the intestinal mucosa. This hypothesis is supported by in vitro studies showing that FXR activation decreased DSS-induced detachment of human enterocyte-like Caco-2 cells from the monolayer but the molecular mechanism was not solved. The demonstration that FXR activation protects against the development of inflammation in murine models of colitis, is consistent with the fact that FXR is highly expressed by the intestinal mucosa and regulates the release of FGF15, which in turn modulates bile acid homeostasis and composition of intestinal microbiota. These results suggest that FXR might represent a novel therapeutic target in inflammatory bowel diseases.

## 7. Conclusions

The data presented in this review illustrate that bile acids are an important component of chemical communications that connect the intestinal microbiota with intestinal immune system. Dysregulated bile acids signaling might be a contributing factor to the development of dysregulated immune response in IBD and might represent an interesting therapeutic opportunity. The two best characterized bile acid receptors, FXR and GPBAR1, are highly expressed in the gastrointestinal tract and exert counter-regulatory action on leukocytes trafficking toward the intestine. According to results from gene knockout mice, it appears that both receptors are essential to maintain a tolerogenic phenotype of intestinal macrophages. Additionally, novel families of bile acids including several oxo-derivatives are emerging as new players in the regulation of intestinal immunity by modulating the activity of RORγt. These oxo-derivatives of bile acids act as inverse agonists for RORγt, exerting a direct anti-inflammatory action, in particular, on Th17 cells, and works are in progress to identify RORγt reverse agonists derived from bile acid to target intestinal inflammation. Finally, there are several approaches that could be used for modulation bile acid pool as well as intestinal FXR, GPBAR1, and RORγt by harnessing the intestinal microbiota by using probiotics or fecal microbial transplantation.

In conclusion, bile acids and their receptors are an essential component of the chemical communications between the intestinal microbiota and the host immune system. Altered bile acid pool impacts on intestinal homeostasis and promotes the immune dysfunction in IBD, making bile acid receptors an interesting therapeutic target in these pathologies.

## Data Availability

Not applicable.
